# Oblique-ilioischial plate technique: a novel method for acetabular fractures involving low posterior column

**DOI:** 10.1186/s12891-022-05487-3

**Published:** 2022-06-06

**Authors:** Zhong Chen, Zhao-xiang Wu, Ge Chen, Yi Ou, Hong-jie Wen

**Affiliations:** 1 Department of Orthopaedic and Trauma, The Second People’s Hospital of Yunnan Province, Kunming, China; 2 Department of Orthopaedic and Trauma, The Affiliated Hospital of Yunnan University, No.176 Qingnian Road, Wuhua District, Kunming City, 650021 Yunnan Province China

**Keywords:** Acetabular fracture, Anterior fixation, modified Stoppa approach, Oblique-ilioischial plate

## Abstract

**Background:**

Complex acetabular fractures involving the anterior and posterior columns are an intractable clinical challenge. The study investigated the safety and efficacy of oblique-ilioischial plate technique for acetabular fractures involving low posterior column.

**Methods:**

A retrospective analysis of 18 patients operated with the oblique-ilioischial plate technique by the modified Stoppa approach (or combined with iliac fossa approach) between August 2016 and July 2021 for low posterior column acetabular fractures was conducted. The anterior column was fixed with a reconstructed plate from the iliac wing along the iliopectineal line to the pubis. The low posterior column was fixed with the novel oblique-ilioischial plate running from the ilium to the ischial ramus. Operative time, intraoperative blood loss, reduction quality, and postoperative hip function were recorded.

**Results:**

Out of the 18 patients, 10 were male and 8 were female. The mean age was 48.6±10.2 years (range: 45–62 years); The mean interval from injury to operation was 7.2±1.4 days (range: 5–19 days); The mean operative time was 2.1±0.3 h (range: 1.0–3.2 hours); The mean intraoperative blood loss was 300±58.4 mL (range: 200–500 mL). Postoperative reduction (Matta’s criteria) was deemed as excellent (*n* = 9), good (*n* = 4), and fair (*n* = 5). At the final follow-up, the hip function (modified Merle d’Aubigne-Postel scale) was deemed as excellent (*n* = 11), good (*n* = 3), and fair (*n* = 4). The mean union time was 4.5±1.8 months (range: 3–6 months). No implant failure, infection, heterotopic ossification, or neurovascular injury were reported.

**Conclusion:**

The oblique-ilioischial plate technique via anterior approach for acetabular fractures involving low posterior column offers reliable fixation, limited invasion, little intraoperative bleeding, and fewer complications. However, larger multicenter control studies are warranted.

## Background

**Fig. 1:**
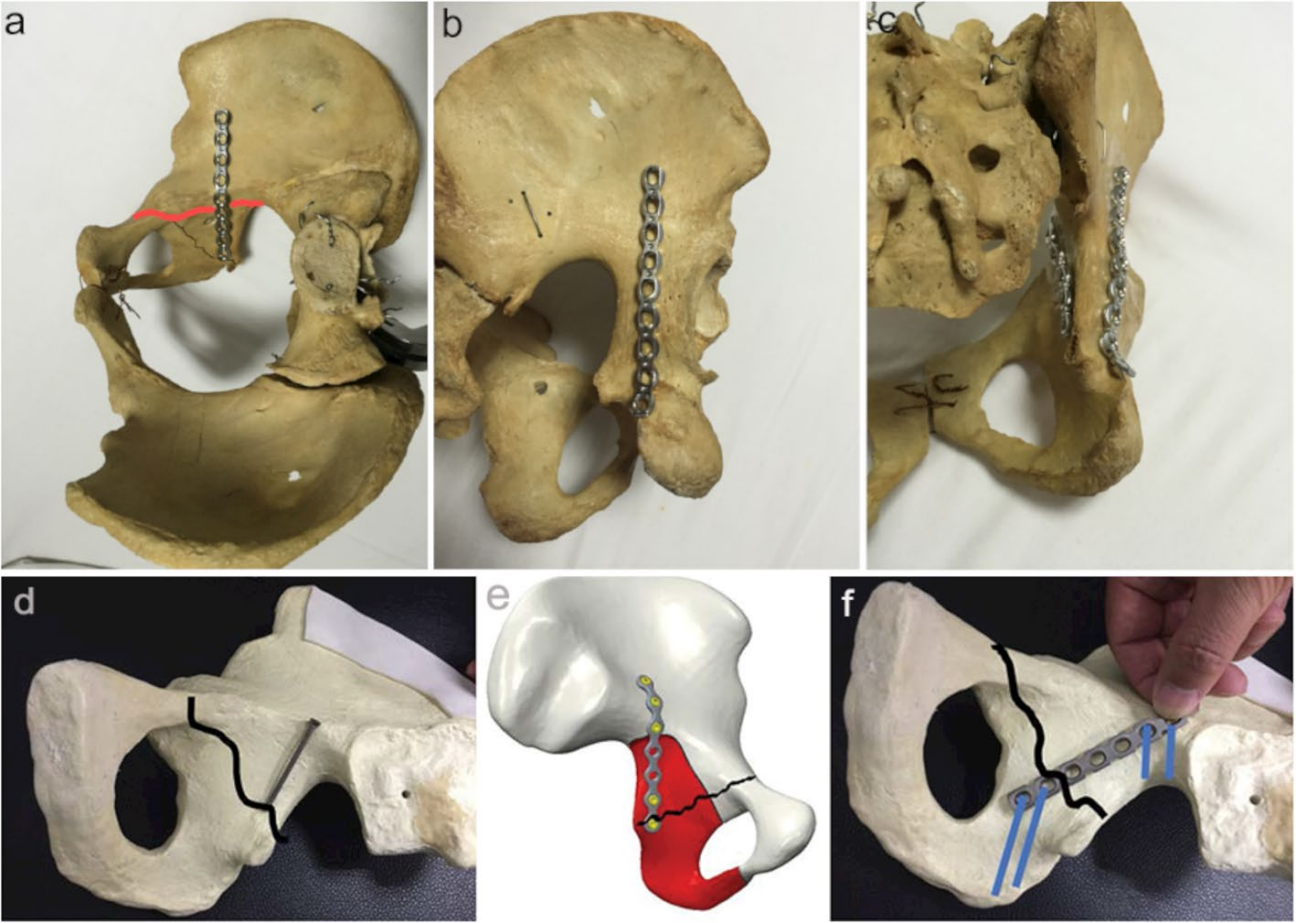
**a-c** Anterior fixation with ilioischial plate for acetabular fractures of posterior column. The projection of ilioischial plate fixation is similar to the posterior plate fixation. **d** Channel screw, **e** ilioischial plate and **f** oblique-ilioischial plate in the fixation of low level posterior column acetabular fracture. Oblique-ilioischial plate technique is obviously superior to the others

A complex acetabular fracture involving both anterior and posterior columns is an intractable clinical problem [1–4]. Consequently, it is a matter of persistent pursuit by all surgeons while treating acetabular fractures to achieve satisfactory outcomes with minimum surgical trauma. With improved understanding and application of the modified Stoppa approach, we introduced an innovative ilioischial plate technique into clinical practice in a previous research series [5] (Fig. 1a-c). Meanwhile, fixation techniques such as the channel screw have also been developed [6–8], which allow the regular use of a single anterior approach to treat an acetabular fracture involving both anterior and posterior columns.

However, fixation of the posterior column with an ilioischial plate, channel screw, or posterior plate is only suitable when the fracture line of the posterior column is high enough [5]. According to our experience, it is difficult to obtain sufficient working length with the ilioischial plate and posterior column screw to achieve stability to treat low acetabular posterior column fractures. Therefore, a posterior approach must also be applied to facilitate fracture fixation. However, it is associated with increased surgical trauma and probability of complications such as ectopic ossification and sciatic nerve injury. Therefore, we created an oblique-ilioischial plate fixation technique to treat low acetabular posterior column fractures through the anterior approach. This technique outlines the reduction and fixation of a low acetabular posterior column fracture with an oblique placement of the reconstructed plate using the modified Stoppa approach.

In this study, we present a retrospective analysis of 18 patients with acetabular fractures involving low posterior column treated by the technique at The Affiliated Hospital of Yunnan University.

## Methods

### Study population

Medical records of patients undergoing oblique-ilioischial plate fixation for low posterior column acetabular fracture between August 2016 and July 2021 were analyzed. Patients with fresh acetabular fractures involving the low posterior column (fracture line is anteriorly-high and posteriorly-low, and fracture line of the posterior column extends to the level of the ischial spine or even farther) were included in the study (Fig. 1d-f). Those with old fractures, fracture of the posterior wall, low fracture line in both anterior and posterior columns (fixation was usually not necessary), and those not suitable for the modified Stoppa approach were excluded.

The study was reviewed and approved by the Ethics Committee of the affiliated hospital at Yunnan University (Kunming, Yunnan, China) and all eligible patients provided written informed consent.

### Procedure

The study included 18 patients who were classified according to the Letoumel-Judet classification [9]. Preoperative X-ray images of the pelvis anteroposterior view, oblique view of the injured iliac bone, and oblique view of the obturator were obtained. Additionally, pelvic computed tomography images and 3D reconstruction data were used to evaluate the fracture type and facilitate surgical approach selection. A single modified Stoppa approach with or without an iliac socket to fix the acetabular fracture involving low posterior column using an oblique-ilioischial plate extending from the iliac bone to ischial ramus was used on the inner side of the pelvis. A combined iliac fossa approach was not required unless there is a high-level anterior column fracture. A combined posterior K–L approach was necessary if it is difficult to achieve a satisfactory outcome through closed reduction and fixation in the posterior wall fracture. The fracture reduction outcomes were rated via the modified Matta standard [10], and hip joint function was assessed using the modified Merle d’Aubigné–Postel score [11]. The quality of fixation was evaluated intraoperatively with the stress test introduced by Firoozabadi [12].

### Surgical method

The surgical procedure used was similar to the original ilioischial plate technique described in our previous study [5]. With the patient in the supine position, we sterilized the surgical area and applied a sterile skin film and bandages to wrap the thigh, calf, and foot and loosen up the iliopsoas muscle and the accompanying neurovascular bundle when the ipsilateral iliopsoas and knee joint are flexing during operation. We then made a longitudinal incision of 8–12 cm starting 2 cm below the umbilicus to 2 cm above the symphysis pubis. Then, the skin, subcutaneous tissue, and the albus abdominis were cut to expose the posterior pubic space, and blunt separation was conducted from the extraperitoneal area. The abdominal hook was used to pull the peritoneum backward and medial, while the abdominal wall together with the external ilioischial artery and vein were pulled outward, and the anterior column, anterior wall, posterior column, and tetrahedron area were revealed [13]. In this procedure, the lateral head of the rectus abdominis muscle may be lifted outward below the periosteum to expose the surgical area. The “death crown” vessel, a branch connecting the obturator vessels to the external ilioischial vessels, should be carefully probed for in the middle part of the superior pubic ramus. If the “dead crown” vessels are present, both severed ends of vessels need to be rigorously ligated.

We cut the periosteum along the arcuate line to expose the fracture ends. Then, the obturator artery, vein, and nerve were retracted inward and protected, and the posterior margin of the obturator ring, posterior margin of ischium, and base of ischium were explored. Finally, we designed the placement of plates and screws according to fracture lines and retracted the external ilioischial artery and vein forward and upward to connect with the ilioischial fossa and expose the ala of the ilium. After reducing the anterior column, a Kirschner wire was used for temporary fixation, and after achieving a reduction of the posterior column fracture, plates and screws were used to fix the posterior column finally. Thus, we were able to avoid the effects of anterior column fracture without anatomic reduction on posterior column fracture reduction. Moreover, since the posterior column fracture is usually inwardly rotationally displaced, we could use tools such as a crowbar to push the fracture segment outward to assist reduction. Additionally, the multiplanar rotational displacement needed to be corrected with caution. Finally, the posterior column was fixed vertically across the fracture line extending from the ilioischial crest to the ischium base with two screws at the distal and proximal ends (Fig. 1f). Nonlocking reconstruction plates were applied and suitably molded to fit the bone surface from the arcuate line to the ischial tuberosity (perpendicular to the fracture line). No special drill was needed to insert the distal screws. The reduction of the medial acetabulum was visually confirmed, and the reduction of the lateral acetabulum was determined by C-arm fluoroscopy. The quality of fixation was evaluated intraoperatively with the stress test.

The protection of blood vessels and nerves was an important consideration. Excessive pulling of the obturator vessels and nerves was avoided, and sufficient space was obtained by blocking them outwards with retractors. Preventing injury to the pelvic venous plexus was also an important consideration. In our experience, placing the first or second proximal screw may stabilize the plate and facilitate the placement of the distal screws. The distal screws were placed under the obturator nerves and vessels with a mean length of 20–30 mm, and the insertion direction was away from the acetabulum and toward the ischial tuberosity.

### Postoperative treatment

After surgery, the affected limb was placed in a slightly elevated position with appropriate flexion of the hip and knee. In this way, soft tissue tension can be reduced and pain can be alleviated. When performing incision closure, the reconstruction of the rectus abdominis at the attachment point of the symphysis pubis was necessary. Thereafter, routine layer-by-layer closure was conducted. A negative pressure drain was placed in the retropubic space and was removed when the drainage fluid was less than 50 ml per day. In addition, the period of antibiotic application is 24 to 72 hours. The pain was controlled by intravenous administration of parecoxib and oral administration of celecoxib. Enoxaparin was used to prevent deep vein thrombosis. Moreover, the positive and passive function exercise of lower extremities after surgery was performed to conferred physical venous thromboprophylaxis. At 3-7 days postoperatively, the patient began to walk with crutches without weight on the affected limb. Patients began partial weight-bearing at 6 weeks and full weight-bearing at 12 weeks.

## Results

Out of the 18 patients, 10 were male and 8 were female, with a mean age of 48.6±10.2 years (range: 45–62 years). Twelve patients sustained a complex acetabular fracture from traffic injuries, whereas the other 6 sustained fractures due to falls from a height. As Letoumel-Judet classification, there were 2 cases of transverse fracture, 6 cases of T-shaped fracture, 3 cases of anterior and posterior hemitransverse fracture, and 4 cases of double-column fracture. Seven cases were of the left hip and 11 cases of the right. The mean time from injury to surgery was 7.2±1.4 days (range: 5–19 days) (Table 1). We observed stable fixation in each case as tested intraoperatively by stress tests. The mean operative time was 2.1±0.3 h (range: 1.0–3.2 hours); The mean intraoperative blood loss was 300±58.4 mL (range: 200–500 mL). The fracture reduction outcomes were rated as the modified Matta standard: 9 cases as excellent, 4 cases good, and 5 cases fair.

**Table 1:** Demographic and baseline data of the study patients

Characteristic		Data)
Sex, n (%)	Male	10(56)
	Female	8(44)
Age, y, mean (range)		48.6(45-62)
Affected side, n (%)	Left	7(39)
	Right	11(61)
Fracture type, n (%) (Le-toumel-Judet classification)	Two-column fx.	4(22)
	Anterior and posterior hemitransverse fx.	6(33)
	T-shaped fx	6(33)
	Transverse fx	2(22)
Interval between injury and surgery, days, mean (range)		7.2(5-19)
Injury type, n (%)	Traffic accident	12(67)
	High falling	6(33)
Approach, n (%)	Modified Stoppa approach	13(72)
	Modified Stoppa approach combined with iliac fossa approach	5(28)

**Fig. 2:**
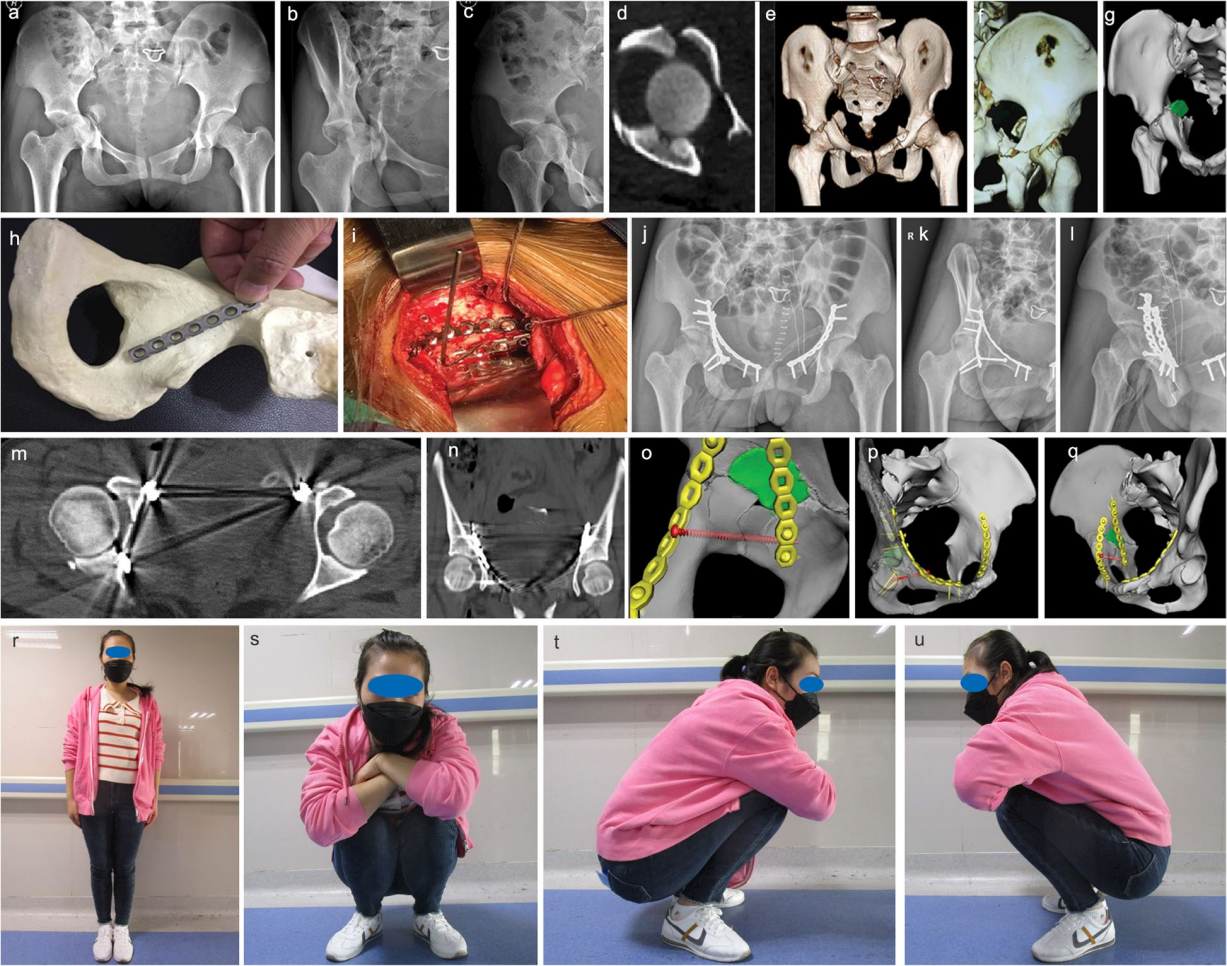
**a**–**c** X-rays of Case no. 1, who was involved in a traffic accident resulting in a right T-shaped acetabular fracture; **d**–**g** the free fragment of “square zone” and low level posterior column fracture line were demonstrated in three-dimensional reconstruction radiograph; **h** preoperative design of the placement of oblique-ilioischial plate; **i** reduction and fixation were performed with the oblique-ilioischial plate through a single modified Stoppa approach. Postoperative radiographs of the pelvis (anteroposterior) **j**, obturator oblique **k**, and iliac crest oblique **l** showed a satisfactory reduction of the fracture, and computed tomography images showed a good anatomic reduction of the fracture **m**, **n**. The three-dimensional reconstruction shows the location of the implant **o**–**q**. Photographs at 9-months postoperatively show that satisfactory hip function was achieved

**Fig. 3:**
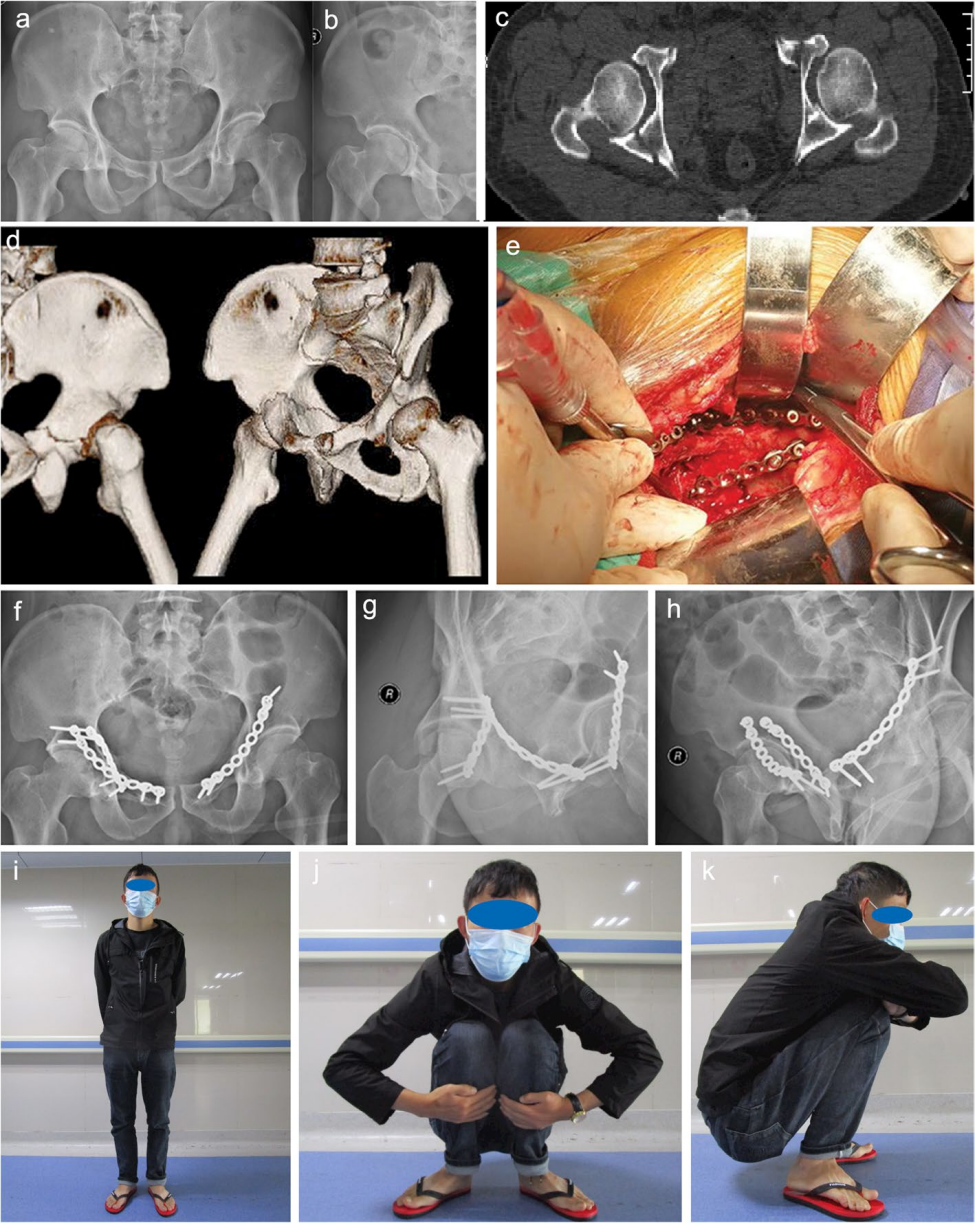
**a**, **b** X-rays of Case no. 2, who was involved in a fall from height resulting in a right T-shaped acetabular fracture combined with pelvic fracture. **c** The low level posterior column fracture line was evident in the plain and three-dimensional reconstruction computed tomography images. **d** Reduction and fixation were performed using the oblique-ilioischial plate through a single modified Stoppa approach. Postoperative radiographs of the anteroposterior view of the pelvis **e**, obturator oblique **f**, and iliac crest oblique **g** views showed a satisfactory reduction of the fracture **m**, **n**. The three-dimensional reconstruction shows the location of the implant **o**–**q**. Photographs at 9-months postoperatively show that satisfactory hip function was achieved

All 18 patients were followed up completely after surgery, and the mean follow-up time was 7.4±1.2 months (range: 6–9 months). Fracture union was achieved in all 18 patients within a mean union time of 4.5±1.8 months (range: 3–6 months). At the last follow-up, the hip joint function was assessed using the modified Merle d’Aubigne-Postel score: 11 cases were excellent, 3 cases were good, and 4 cases were fair. No complications such as internal fixation failure, infection, ectopic ossification, or neurovascular injury were observed during follow-up (Table 2). Figures 2 and 3 present pictures of the typical cases.

**Table 2:** Outcome including perioperative parameters

Parameters		Data
Operative time (h), mean (range)		2.1(1.0-3.2)
Intraoperative blood loss (ml), mean (range)		300(200-500)
Reduction assessment (Matta's criteria), n (%)	Excellent	9(50)
	Good	4(22)
	Fair	5(28)
Follow-up (month), mean (range)		7(6-9)
Hip function (Modified Merle d'Aubigne-Postel scale), n (%)	Excellent	11(61)
	Good	3(17)
	Fair	4(22)
Union time (month), mean (range)		4.5(3-6)
Complication, n (%)		0(0)

## Discussion

**Fig. 4:**
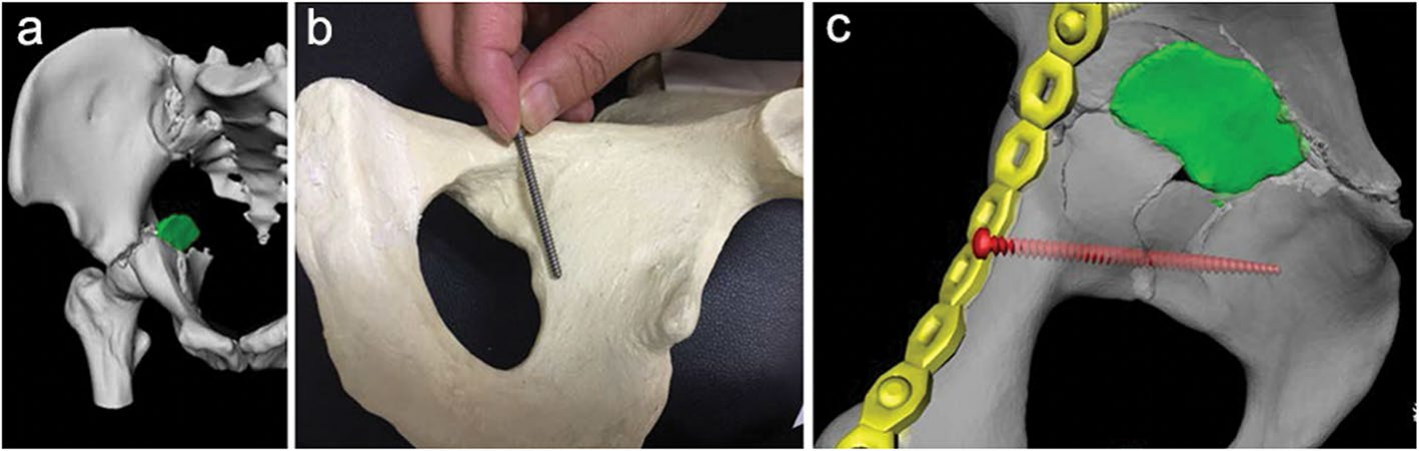
**a**-**c** The pictures show the displacement of posterior column fracture. The fixation of one screw in the “door axis” may not achieve enough stable

Surgical treatment of acetabular fractures involving the anterior and posterior columns remains a challenging task. Over the recent years, ilioischial plate application has become increasingly popular as a treatment option for acetabular posterior column fractures along with posterior plate and posterior column screws [14–17]. However, the three methods are only suitable for acetabular posterior column fracture with a high fracture line. There are several disadvantages when applying a posterior-approach plate for reducing acetabular fractures. First, an additional posterior approach is required. Second, the low fracture line of the posterior column usually hampers posterior plate placement, which requires excessive soft tissue dissection. Additionally, since the displaced posterior column fracture fragment is generally rotating inward, the posterior plate mainly plays a “lift and pull” role on the posterior column fracture fragment, whereas the anterior fixation mainly produces a “push and pressure” force. Hence, anterior fixation is better suited for this scenario as per the biomechanical considerations [10, 18]. The three aforementioned conventional treatment methods also have certain limitations in treating low posterior column fractures. Some authors have described inserting articular screws from the pubic bone to the anterior acetabular edge by the anterior approach to achieve fixation [19]. However, posterior column fracture displacement is not a simple inward movement, but rotational displacement. Consequently, it is difficult to achieve an ideal control of rotation and inward displacement by placing only one screw in the “door axis” position (Fig. 4a-c). We observed that there was sufficient space to place a plate obliquely across the fracture line of the low posterior column through the anterior approach. Furthermore, we found that the placement of oblique-ilioischial plates can be achieved only by exposing the anterior and middle fracture lines and the acetabular quadrangular region partially. Besides, this method was easier to perform when compared with the ilioischial plate technique and more conducive to protecting the local blood vessels and nerves.

The methods of reduction and fixation of acetabular fracture are various. Veliceasa et al performed a study of a modified Stoppa approach using an anatomically precontoured plate for the treatment of acetabular fractures, which achieved a very low complication rate and good to excellent results in 89% of the cases [20]. Mitchell et al suggest that it is an effective and feasible method to address transverse acetabular fractures with regularly performed a posterior approach and used a clamp from the back to the front to reduce and hold in position the anterior column before the final fixation with screws [21]. Additionally, two studies provided important biomechanical fixation evidence of anterior column posterior hemitransverse and transverse types acetabular fracture by vitro model test [22]. However, those studies did not specifically focus on the problem of low posterior column fracture fixation. In our series, the operation time, intraoperative blood loss, postoperative scores, and union time showed no significant differences with previous literature [20, 23, 24]. This suggests that the oblique-ilioischial plate technique is a practical and effective option for acetabular low posterior column fracture, especially in the case of acetabular fractures involving both anterior and posterior columns. Because of our lack of experience in the early stages of the application of this technique, the prebending of the oblique–ilioischial plate was inaccurate. Additionally, the screw was a nonlocking system, which led to the loss of fracture reduction after screw tightening and unsatisfactory results. We hope to develop an anatomical locking plate system that can effectively solve the problem of secondary reduction and loss of fracture caused by insufficient prebending plate and screw compression. When applying the oblique-ilioischial plate technique, it is necessary to master the indications of oblique-ilioischial plate technique: i) fresh acetabular fracture involving posterior column; ii) complex acetabular fracture involving the posterior column; iii) transverse fracture of the acetabular posterior column without the posterior wall. Additionally, a prerequisite as our study is that the case is suitable for the modified Stoppa approach.

There were several limitations to this study. First, the sample size was small and the follow-up duration was short. Second, the lack of a biomechanical analysis underlies the insufficient evidence supporting the superiority of the method. We also lacked sufficient experience in screw insertion in the posterior acetabular column, and we concluded that the general direction of the screw should be toward the ischial tubercle as much as possible to hold sufficient bone mass and obtain an ample insertion angle to avoid the articular cavity.

In conclusion, the oblique-ilioischial plate technique via anterior approach may be a good treatment option for acetabular fractures involving low posterior column as it offers the advantages of reliable fixation, limited invasion, little intraoperative bleeding, and fewer complications. However, multicentric prospective control studies with larger samples are warranted to prove the safety and efficacy of the method.

## Data Availability

The data used and analyzed during the current study are available in anonymized form from the corresponding author on reasonable request.
